# Locomotive syndrome: history, current concepts, and future perspectives

**DOI:** 10.1007/s00264-026-06983-0

**Published:** 2026-08-19

**Authors:** Takashi Ohe

**Affiliations:** 1 Department of Orthopaedics, NTT Medical Center Tokyo, Shinagawa-ku , Tokyo, Japan; 2 Locomo Challenge! Collective, Bunkyo-ku, Tokyo, Japan

**Keywords:** Locomotive syndrome, Mobility, Locomotive syndrome Risk Test, Locomotive syndrome Stage

## Abstract

This review outlines the concept of locomotive syndrome, as well as the current definition, diagnostic criteria, and management strategies for the condition. This paper begins by discussing the reasons behind the introduction of the concept of locomotive syndrome in Japan and its social context. This social context is characterized by Japan’s population aging at the fastest rate in the world, changes in its industrial structure leading to a decrease in traumatic injuries such as workplace accidents, and a sharp increase in degenerative diseases. The prevalence of a relatively small number of musculoskeletal disorders is high among older adults, and the rate of co-morbidity is also high. Furthermore, these disorders and conditions are interlinked through a decline in motor function, such as reduced muscle strength and impaired balance. As multiple disorders and conditions often co-exist in a single older adult, their combined effect leads to a decline in mobility. The coexistence and interaction of these disorders are defining characteristics of musculoskeletal diseases in older adults and represent a major clinical challenge. The concept of locomotive syndrome was proposed precisely because it is necessary to consider such musculoskeletal problems in older adults comprehensively from the perspective of reduced mobility. Subsequently, the locomotive syndrome risk test and the locomotive syndrome stage are explained. Following this, locomotion training is introduced as a countermeasure against locomotive syndrome. Finally, this paper discusses the relationship between locomotive syndrome and other fields, such as cancer and metabolic syndrome, as well as future perspectives.

## Introduction

I would like to begin by describing the experience that ultimately led to the development of the concept of Locomotive syndrome. Beginning in 2000, I began to notice a change in the types of injuries and illnesses, as well as the age distribution, of patients being admitted to the emergency hospital where I work. Traumatic injuries among young and middle-aged people resulting from workplace accidents and road traffic accidents had been declining rapidly. At the same time, the age profile of patients with musculoskeletal conditions was rapidly shifting towards older age groups, and the range of conditions they presented with was changing rapidly as a result. Recurrent ambulance admissions of older adults with multiple musculoskeletal disorders became increasingly common. Many older adults with multiple musculoskeletal conditions had ended up requiring long-term care. I felt that the traditional orthopedic approach, which primarily focused on repairing individual lesions, was insufficient for addressing this emerging problem. I conveyed this concern to Professor Kozo Nakamura of the Department of Orthopaedic Surgery at the University of Tokyo, who was then President of the Japanese Orthopaedic Association (JOA), and explained that a new concept or term might be needed to describe this phenomenon. Subsequently, Professor Nakamura conducted an in-depth analysis of the changes in the disease profile brought about by shifts in Japan’s demographic structure and societal changes at the time. In 2007, he introduced the concept of Locomotive syndrome, and in 2008, he published the first concise explanation of Locomotive syndrome [1], thereby laying the foundations for the JOA to carry out ongoing research and awareness-raising activities regarding the syndrome [2]. In Japan, the term ‘LOCOMO’ has been adopted for Locomotive syndrome to make it more recognizable to the public.

### 1) The reasons for proposing the concept of locomotive syndrome in Japan, and the social context changes in Japan’s demographic structure

Population ageing has progressed far more rapidly in Japan than in most Western countries.

The period it took Japan to transition from an “aging society” to an “aged society” as defined by the WHO—that is, for the proportion of older adults to double from 7 to 14%—was only 24 years, from 1970 to 1994. This is far shorter than the 40 years for Germany, 46 years for the United Kingdom, 61 years for Italy, 72 years for Australia, 85 years for Sweden, and 115 years for France. This rapid demographic transition brought age-related healthcare challenges into sharp focus. It was clear that the sharp rise in the proportion of the older adults would lead to a sharp increase in the diseases they suffer from and a sharp rise in demand for medical care.

## Changes in Japan’s industrial structure

The share of the workforce employed in manufacturing as a percentage of the total workforce has continued to decline, from 26% in 1970 to 16% in 2010, and is projected to reach 15% by 2024. As a result, the nature of workplace accidents has also changed. Specifically, injuries such as upper limb trauma-typically caused by being pinched or entangled in machinery—have declined rapidly, from 25,000 cases annually in 2000 to 15,000 in 2010. This trend was consistent with the observations made in emergency clinical practice.

## Decline in traffic accident fatalities and injuries in Japan

In Japan, the number of traffic accident fatalities exceeded 10,000 per year from 1988 to 1991, but subsequently declined, falling below 5,000 in 2010—less than half the previous level. The number of traffic accident injuries peaked at 1.18 million in 2004, then declined, falling below 600,000 in 2017—also less than half the previous level. The decline in traffic accident fatalities and injuries was largely due to a significant increase in seatbelt usage rates and improvements in vehicle safety features, such as airbags and ABS, which have become nearly standard equipment. Around the time the concept of Locomotive syndrome was introduced in 2007, the number of seriously injured traffic accident victims was declining rapidly.

## Epidemiological research on musculoskeletal disorders in an ageing population

In this field, Noriko Yoshimura and colleagues at the 22nd Century Medical&Research Centre at the University of Tokyo Hospital have established a cohort of over 3,000 local residents in urban areas of Tokyo and in mountainous and fishing villages in Wakayama Prefecture, and have been conducting a longitudinal survey approximately every three years since 2005[3–5]. This cohort has generated extensive epidemiological evidence regarding the prevalence, risk factors, and comorbidities of musculoskeletal disorders. Her team reported extremely high prevalence rates of osteoporosis, osteoarthritis of the knee and lumbar spondylosis, conditions that are common among middle-aged and older adults. Estimates of the number of people aged 40 and over affected by these conditions indicate that osteoporosis affects 15.9 million people, osteoarthritis of the knee affects 25.3 million people, and lumbar spondylosis affects 37.9 million people. Furthermore, these conditions often co-occur; it has been reported that approximately 30 per cent of women in their 70 s, approximately 10 per cent of men aged 80 and over, and approximately 40 per cent of women aged 80 and over have all three conditions.

## Characteristics of musculoskeletal disorders in older adults

As epidemiological studies show, the prevalence of a relatively small number of musculoskeletal disorders is high among older adults, and the rate of co-morbidity is also high. Furthermore, these disorders and conditions are interlinked through a decline in motor function, such as reduced muscle strength and impaired balance. As multiple disorders and conditions often co-exist in a single older adult, their combined effect leads to a decline in mobility. The coexistence and interaction of these disorders are defining characteristics of musculoskeletal diseases in older adults and represent a major clinical challenge. It is necessary to consider such musculoskeletal problems in older adults comprehensively from the perspective of reduced mobility.

## Reasons for requiring long-term care and musculoskeletal disorders

In Japan, musculoskeletal disorders account for one-quarter of the reasons for requiring long-term care. In 2007, fractures and falls accounted for 11.8 per cent, joint disorders for 11.3 per cent, and spinal cord injuries for 2 per cent, totaling 25.1 per cent; by 2022, however, fractures and falls accounted for 13.9 per cent, joint diseases for 10.2 per cent and spinal cord injuries for 2 per cent, totaling 26.1 per cent. In order to prevent the need for long-term care, preventive strategies were needed to address these musculoskeletal conditions and injuries, which become particularly pronounced in old age.

### 2) Definition and Concept of Locomotive syndrome (LS)

JOA defines "locomotive syndrome" as a condition of reduced mobility due to disorders of the locomotor (musculoskeletal) system—a state that, if it progresses, increases the risk of requiring nursing care.

**Fig. 1:**
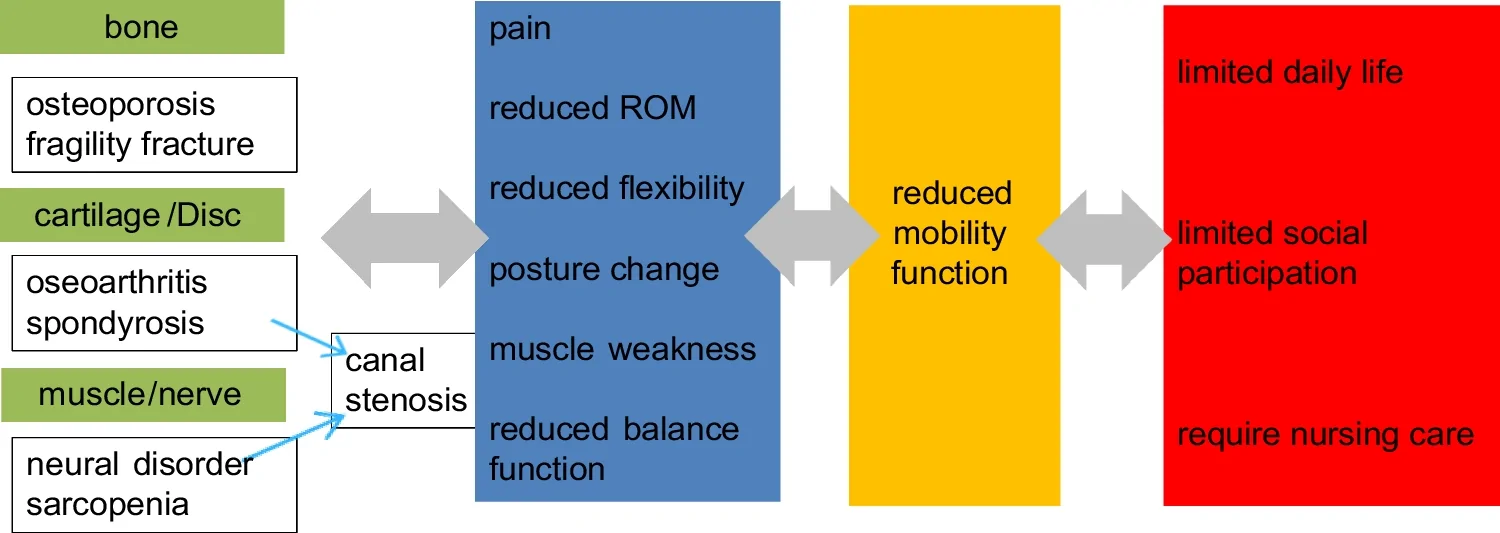
Conceptual diagram illustrating the components of LS

Figure 1 shows a conceptual diagram illustrating the components of LS. Osteoporosis, osteoarthritis, degenerative spondylosis, spinal stenosis and sarcopenia are all common disorders in middle-aged and older adults.

When these musculoskeletal disorders occur, they interact and compound to cause musculoskeletal pain and functional decline, such as reduced muscle strength and impaired balance.

This functional decline, in turn, further exacerbates the musculoskeletal disorders, leading to a deterioration in mobility (gait disturbance); if the condition worsens further, it ultimately results in a state requiring long-term care.

In this conceptual diagram, the conditions shown on the far left are the primary musculoskeletal disorders that cause LS. Please note that, from the outset, the conceptual diagram for LS has distinguished between sarcopenia—a musculoskeletal disorder—and muscle weakness—a musculoskeletal functional impairment. This distinction is often overlooked in clinical practice. Furthermore, as sarcopenia is a systemic condition, it does not include the localized muscle atrophy that we orthopaedic surgeons frequently encounter. Examples of conditions not included in the definition of sarcopenia include localized muscle atrophy caused by pain from osteoarthritis, reversible muscle atrophy following an osteoporotic fracture, or neurogenic muscle atrophy due to nerve compression caused by spinal stenosis are not included in the definition of sarcopenia.

Grip strength, which is used for the diagnosis of sarcopenia, is reduced in cases of osteoarthritis of the hands. Takamoto et al. reported that in individuals with hand disorders, grip strength is reduced to a level below the sarcopenia threshold [6]. We believe this is a point that should be borne in mind when using grip strength as a proxy for overall muscle strength.

### 3) Assessment of LS

LS is assessed using the following three tests, collectively known as the "locomotive syndrome risk tests (LS risk tests)." These tests consist of two physical function assessments and one self-administered questionnaire. Clinical threshold values ​​are established for each of the three tests to classify the severity of LS into four categories: no LS, LS Stage 1, LS Stage 2, or LS Stage 3.

#### Stand-up test

This test evaluates the ability to stand up from seat heights of 10, 20, 30, or 40 cm using one leg or both legs. Developed by Muranaga, this test assesses vertical mobility and shows a strong correlation with the ratio of knee extension strength to body weight [7].

#### Two-step test

This test measures the distance covered in two steps taken using the maximum possible stride length—without losing balance—and divides that distance by the individual's height to calculate the "Two-step test value". Also developed by Muranaga, this test assesses horizontal mobility and correlates well with walking speed [8].

Ogata et al. have reported on the reliability of these two physical function tests, noting the absence of floor or ceiling effects [9].

#### 25-question geriatric locomotive function scale (GLFS-25)

This questionnaire was developed by Hoshino, and colleagues [10]. It comprises 25 items related to the musculoskeletal system; respondents rate the extent to which each item applies to them on a scale of 0 to 4 points, and a total score is calculated. A higher score indicates a greater degree of perceived limitation or difficulty related to the musculoskeletal system. GLFS-25 serves as a subjective indicator of physical condition and lifestyle status; its validity and reliability as a scale have been verified by Seichi et al. [11]. Kawahara et al. have reported on how to handle cases where there are missing responses [12].

The specific procedures for these tests are explained in detail on the Locomo Challenge! Collective’s website (https://locomo-joa.jp/en).

## Significance of the LS risk tests

The stand-up test evaluates overall vertical mobility. As previously mentioned, this test is closely linked to the relationship between body weight and knee extension strength. The results in elderly individuals are also influenced by factors such as pain in lower-limb joints or restricted range of motion.

The Two-step Test evaluates overall horizontal mobility. Although this test correlates well with maximum walking speed, it is also influenced by lower-limb strength for push-off, balance ability required to take two consecutive steps, and lower-limb flexibility needed for a long stride.

Research by Ogata et al. indicates that the interrelationships among the three LS risk tests are weak [8]; therefore, it is recommended that all three tests be performed to assess for LS.

## Assessment of LS stage using LS risk test

In 2015, the JOA established and published categories ​​for "LS Stage 1" and "LS Stage 2" based on the LS risk test. These categories ​​were designed to assess the severity of LS regardless of age—from the perspective of preventive medicine—with the aim of facilitating prevention and halting further deterioration.

This initiative was based on findings from a study [13] examining the relationship between the LS risk tests and known risk factors for requiring long-term care, as identified in population-based cohort studies. The three categories ​​established for the LS risk test in that study were shown to be significantly and independently associated with "slow walking speed" and "long time required to stand up from a chair"—factors previously identified in longitudinal studies as predictors of a transition to requiring long-term care. Furthermore, the strength of this association increased with the number of applicable categories met. Although the LS risk test was introduced relatively recently and no longitudinal studies have yet used it with the transition to requiring long-term care as an endpoint, the JOA—as a body of experts—determined these categories ​​to be valid clinical cutoffs for LS, recognizing the urgent need to establish such standards.

Subsequently, in 2020, the JOA established and published a category for "LS Stage 3," representing a stage of LS progression beyond LS Stage 2. The category for LS Stage 3 was set to be roughly equivalent to the physical functional aspects of the frailty phenotype. The frailty phenotype criteria specify a walking speed of 1.0 m/s. Regarding the relationship between walking speed and the Two-Step Test; a normal walking speed of 1.0 m/s corresponds to a Two-step test value of 0.9. In research concerning the GLFS-25, Iwaya reported that GLFS-25 scores can be categorized into seven levels [14]. According to this classification, the 24–31 point range is the first level where the proportion of individuals experiencing difficulty in four specific areas—rising from a chair, getting dressed, going out using public transportation, and socializing with close friends—exceeds 50%; thus, this range represents a state where "mobility decline has progressed to a level that interferes with social participation."

Classification into "No LS," "LS Stage 1," "LS Stage 2," or "LS Stage 3" is determined based on the results of three LS risk tests (Table 1). LS Stage 1 marks the onset of the condition; LS Stage 2 indicates progressed decline in mobility function; and LS Stage 3 indicates a state where the decline in mobility function has progressed sufficiently to interfere with social life.

**Table 1.**
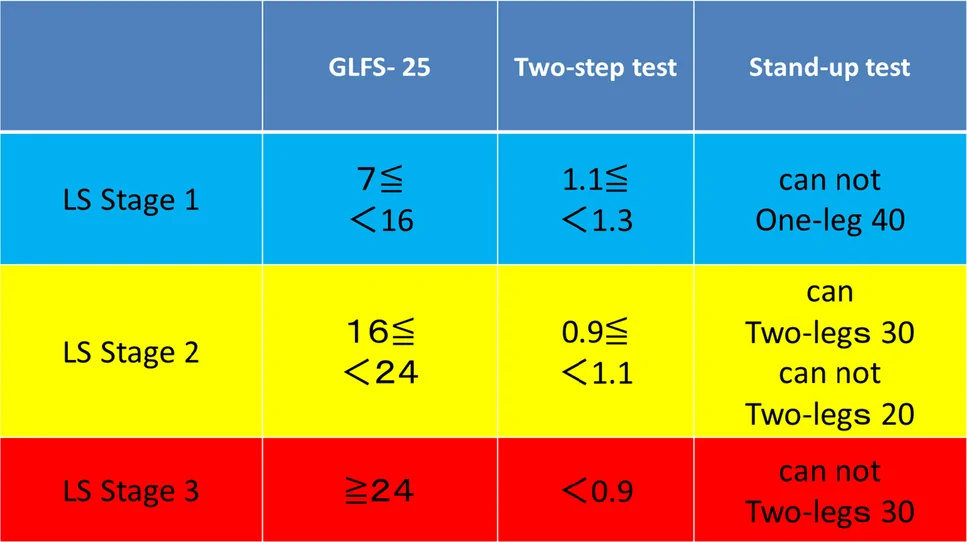
LS Stages based on the results of three LS risk tests

Using this LS Stage category, it is possible to assess the effectiveness of orthopaedic surgeries regardless of the specific procedure performed. It has been reported that, for spinal stenosis surgery, total knee arthroplasty, and total hip arthroplasty the vast majority of patients presented with LS Stage 3 prior to surgery—with two-thirds scoring 24 points or more—while post-operative GLFS-25 scores largely improved to below 24 points. [15–20].

Using this category for LS Stage 3, Yoshimura et al. reported findings from a longitudinal study showing that individuals at LS Stage 3 face a 3.6-fold higher risk of requiring long-term care three years later compared to those without LS [21].

## Reference values for the LS risk test by gender and age group

Between 2017 and 2019, the Locomo Challenge! Promotion Council (Locomo Challenge! Collective) conducted a survey to establish reference values by gender and age group. The survey targeted community-dwelling adults aged 20–29, 30–39, 40–44, 45–49, 50–54, 55–59, 60–64, 65–69, 70–74, 75–79 and 80–89. The aim was to enroll 464 men and women in each age group (totaling 10,208 participants). The target number of participants for each of the seven regions nationwide (Hokkaido, Tohoku, Kanto, Chubu, Kinki, Chugoku/Shikoku and Kyushu) was set based on population proportions, and the survey was conducted face-to-face. (This research was conducted in accordance with the ‘Ethical Guidelines for Life Science and Medical Research Involving Human Subjects’ established by the Japanese Government, and written informed consent was obtained from the participants. Furthermore, this study was approved by the Ethics Committee of the JOA.) Using this data, Yamada et al. analyzed 8,681 individuals (3,607 men and 5,074 women) and reported their findings [22]. Furthermore, the Locomo Challenge! Promotion Council has published a graph incorporating these results on its website.

## Number of people with locomotive syndrome and the number by severity stage

Research by Yoshimura et al. has reported the number of individuals classified by LS stage. According to these findings, as of 2015, the estimated prevalence of individuals meeting LS Stage 1 or higher was approximately 46.6 million; for Stage 2 or higher, 16.4 million; and for Stage 3, 7.1 million [21].

### 4) Prevention and management of LS

If a person is known to have a disorder of the locomotor (musculoskeletal) system (as shown in the conceptual diagram), treating that disorder serves as a measure against LS.

For individuals who experience a decline in mobility function but do not yet suffer from a specific disorder, in 2010 the JOA has introduced "Locomotion Training" (LT) as a preventive and management strategy. LT can be performed at home without special equipment; it consists of just two types of exercises that are easy to learn and quick to complete. Furthermore, LT is designed so that it can be adapted to the severity of an individual's condition.

## LT #1: Single-leg standing with eyes open

This is a simple exercise involving standing on one leg. It is, in itself, a method of measuring physical fitness, specifically balance. To avoid the risk of falling, participants are encouraged to perform this exercise near something they can hold onto, or to support themselves with their hands or fingers. As research by the JOA has shown that one minute of training is effective in preventing falls [23], the target is to maintain this position for one minute. Even for those who cannot manage more than one minute without support, they should be encouraged to stand for one minute whilst supporting themselves with their hands or fingers.

## LT #2: Squats

The second LT is the squat. It is best to perform this exercise with the sensation of lowering your bottom rather than simply bending your knees. Ensure that your kneecaps do not extend beyond your toes, and squat whilst bending your knees in the direction of your second toe. Bend your knees only slightly; do not bend them to a right angle or beyond. Perform the exercise whilst engaging both the quadriceps (the knee extensors) and the hamstrings (the knee flexors) simultaneously. Keep each session short, doing just five to six repetitions at a time, and aim to perform them frequently, at least three times a day. Squats also strengthen muscles close to the body’s centre, such as the gluteal muscles and the psoas major.

Since the report by Ishibashi et al. [24, 25], there have been numerous reports that LT improves motor function in all groups, including residents of care homes, older people living in the community and working people [26–29].

### 5)Research and awareness-raising activities concerning the relationship between LS and other medical fields

## LS in cancer patients

In Japan, more than one in two people will develop cancer at some point in their lives. Consequently, the number of patients living with cancer continues to rise. In such patients, LS may develop as a result of the cancer itself or its treatment. Three major mechanisms have been proposed: musculoskeletal problems caused by the cancer itself; musculoskeletal problems arising from cancer treatment; and musculoskeletal disorders co-existing with the cancer [30, 31]. A report by Morioka et al., summarizing a questionnaire survey conducted by the JOA, revealed the low level of involvement of orthopaedic surgeons in cancer care [32]. There are still numerous cases where musculoskeletal management of cancer patients is not being carried out appropriately, and Kawano et al. highlight this as a challenge for orthopaedic surgeons in the future [33].

## LS and metabolic syndrome (MS)

Research into the relationship between LS and metabolic syndrome is advancing. In Japan, nationwide health screening programs are widely available, with costs supported by local authorities and health insurance associations. These screenings include comprehensive clinical tests designed for the early detection of atherosclerosis, a primary risk factor for cardiovascular disease and stroke.

Nakagawa et al. analyzed large-scale, real-world data from 35,059 individuals who underwent these health check-ups, reporting that those with MS had a 1.87-fold higher risk of developing LS compared to those without MS [34]. Utilizing this dataset, Yamaguchi et al. demonstrated in longitudinal study that both a decline in physical function and a lack of exercise habits significantly increase the incidence of both LS and MS [35, 36].

Yoshimura et al. conducted an epidemiological study on the relationship between musculoskeletal disorders, which constitute LS, and visceral disorders, which constitute MS, and reported a correlation between obesity, hypertension, impaired glucose tolerance and osteoarthritis of the knee [37]. A meta-analysis on the relationship between hypertension and osteoarthritis also highlighted differences depending on the joint site [38]. With regard to the relationship between LS and MS, it is possible that systemic chronic inflammation plays a role, rather than the effects of weight gain due to obesity alone.

## Conclusion

The term and concept of LS came into being because Japan became the world’s first super-aged society, a development that coincided with changes in the social environment, including industry and transport. Over the subsequent twenty years, a body of knowledge on LS has been accumulated. The accumulated evidence on LS may provide valuable insights for countries and regions facing rapid population aging and increasing burdens of musculoskeletal disease.

As longevity continues to increase worldwide, the concept of the LS provides orthopaedic surgeons with a practical framework for identifying declines in mobility, stratifying risk, and implementing preventive intervention in aging societies.

## Data Availability

No datasets were generated or analysed during the current study.
